# Rapid comparative evaluation of SARS-CoV-2 rapid point-of-care antigen tests

**DOI:** 10.1007/s15010-022-01810-1

**Published:** 2022-04-09

**Authors:** Anna Denzler, Max L. Jacobs, Victoria Witte, Paul Schnitzler, Claudia M. Denkinger, Michael Knop

**Affiliations:** 1 Center for Molecular Biology of Heidelberg University (ZMBH), Heidelberg University, Heidelberg, Germany; 2 Department of Infectious Diseases, Virology, Heidelberg University Hospital, Heidelberg, Germany; 3 Department of Infectious Diseases, Division of Infectious Disease and Tropical Medicine, Heidelberg University Hospital, Heidelberg, Germany; 4 German Cancer Research Center (DKFZ), Heidelberg, Germany; 5 DKFZ-ZMBH Alliance, Heidelberg, Germany

**Keywords:** Comparison of antigen-detecting rapid diagnostic tests, COVID-19, SARS-CoV-2, Test sensitivity, Non-functional AgPOC tests

## Abstract

**Purpose:**

The objective of this study was to develop a scalable approach for direct comparison of the analytical sensitivities of commercially available SARS-CoV-2 antigen point-of-care tests (AgPOCTs) to rapidly identify poor-performing products.

**Methods:**

We present a methodology for quick assessment of the sensitivity of SARS-CoV-2 AgPOCTs suitable for quality evaluation of many different products. We established reference samples with high, medium, and low SARS-CoV-2 viral loads along with a SARS-CoV-2 negative control sample. Test samples were used to semi-quantitatively assess the analytical sensitivities of 32 different commercial AgPOCTs in a head-to-head comparison.

**Results:**

Among 32 SARS-CoV-2 AgPOCTs tested, we observe sensitivity differences across a broad range of viral loads (9.8 × 10^8^ to 1.8 × 10^5^ SARS-CoV-2 genome copies per ml). 23 AgPOCTs detected the Ct25 test sample (1.6 × 10^6^ copies/ml), while only five tests detected the Ct28 test sample (1.8 × 10^5^ copies/ml). In the low-range of analytical sensitivity, we found three saliva spit tests only delivering positive results for the Ct21 sample (2.7 × 10^7^ copies/ml). Comparison with published data supports our AgPOCT ranking. Importantly, we identified an AgPOCT widely offered, which did not reliably recognize the sample with the highest viral load (Ct16 test sample with 9.8 × 10^8^ copies/ml) leading to serious doubts about its usefulness in SARS-CoV-2 diagnostics.

**Conclusion:**

The results show that the rapid sensitivity assessment procedure presented here provides useful estimations on the analytical sensitivities of 32 AgPOCTs and identified a widely-spread AgPOCT with concerningly low sensitivity.

**Supplementary Information:**

The online version contains supplementary material available at 10.1007/s15010-022-01810-1.

## Introduction

In the SARS-CoV-2 pandemic, lateral flow antigen tests developed as a rapid alternative to SARS-CoV-2 reverse transcriptase quantitative polymerase chain reaction (RT-qPCR)-based diagnostics. Because of their ease of use, lateral flow antigen tests are applicable for point-of-care (POC) as well as self-testing and can, therefore, be incorporated in daily life to support viral containment [1]. In the following, these tests will be referred to as antigen point-of-care tests (AgPOCTs). AgPOCTs are meanwhile widely used for SARS-CoV-2 diagnostics and screening purposes. Currently, several hundred different SARS-CoV-2 AgPOCT brands are commercially available to meet the demand (545 products for professional use are listed by the Federal Institute for Drugs and Medical Devices (Bundesinstitut für Arzneimittel und Medizinprodukte (BfArM)); as of July 27, 2021; [2]). However, the sensitivity and specificity of these are not systematically assessed.

If an AgPOCT is used by a professional operator, it falls under the *low-risk* category of the European Union directive on In Vitro Diagnostics (IVD), which currently governs marketing authorization for IVD in Europe. Under this directive, manufacturers can still self-certify SARS-CoV-2 AgPOCTs and waive independent quality control before these products enter the market. The validation of available AgPOCTs is, therefore, not assured in the view of the Paul Ehrlich Institute (PEI), Federal Institute for Vaccines and Biomedical Products, Germany. Furthermore, the PEI reports on evidence of counterfeiting. A new regulation governing independent validation by specialized and certified reference laboratories is planned, but will only become effective in May 2022 at the earliest (https://www.pei.de/DE/newsroom/hp-meldungen/2020/200323-covid-19-nat-tests.html;jsessionid=F786872EBB85959AE8DA2B8FCB3ABE00.intranet222?nn=169730).

If an AgPOCT is distributed for layperson use, it falls under a *higher-risk* category and requires independent validation. This validation of sensitivity is currently performed by the PEI together with reference laboratories and a list with examined AgPOCTs passing their criteria is provided [3]. AgPOCTs failing the comparative evaluation by PEI will be removed from the list provided by the BfArM [2]. This list, however, comprises only products from manufacturers or distributors, which also registered their products for listing (https://www.bfarm.de/DE/Medizinprodukte/Aufgaben/Spezialthemen/Antigentests/_node.html), rendering the absence of an AgPOCT from this list difficult to interpret.

Many in-depth AgPOCT characterization studies show that AgPOCT sensitivities can vary substantially. One study reporting on the validation of 122 AgPOCs was recently published [4]. The authors found that 26 AgPOCTs do not fulfill the required minimum sensitivity, clearly illustrating that many circulating AgPOCTs are insufficiently sensitive. In addition to this, significant brand-to-brand and lot-to-lot variations were observed [5]. These circumstances urge the need for an easy-to-use method to quickly assess AgPOCTs at market entry and periodically thereafter for post-implementation quality control.

In this study, we sought to establish a procedure to rapidly evaluate the sensitivity of a large number of AgPOCTs using a small test sample panel and several tests per product. To this end, we developed a strategy involving pooled samples and four different dilution steps from high to low viral loads and generated several hundred aliquots thereof. Using this approach we then investigated 32 AgPOCTs, which included mainly tests currently in use in the local area (Heidelberg, Germany). We compared the results with data from the literature, which enabled us to conclude the validity of our approach and the performance of the products investigated.

## Methods

### Study design

We tested the analytical sensitivity of a large number of commercially available AgPOCTs by applying pooled samples from nasopharyngeal swabs with defined SARS-CoV-2 viral loads (given in cycle threshold (Ct)) including Ct16, Ct21, Ct25, and Ct28 (~ 9.8× 10^8^ to ~ 1.8 × 10^5^ genome copies per ml) as well as a pooled sample obtained from SARS-CoV-2 negative tested persons. Pools were generated using anonymized remnant swab sample material collected for the clinical diagnosis of a SARS-CoV-2 infection by RT-qPCR carried out by the Center for Infectious Diseases, Virology, Heidelberg University Hospital, Germany. Pharyngeal swab specimens were collected through the nose (nasopharyngeal) and contained in viral transport medium (VTM). Per test, 50 µl of the samples were mixed with the provided lysis buffer of each AgPOCT, and the tests were performed strictly according to the manufacturer’s instructions. After the recommended incubation time, we acquired images of the test chambers using a Panasonic Lumix DMC-G70 camera equipped with a Panasonic H-FS12060 objective. We tested AgPOCTs at least in duplicates with the corresponding test samples. We quantified test results by measuring the background-corrected signal intensities of the test (T) band versus control (C) band in ImageJ (v1.53c) using the *Gels* analysis function usually used for quantification of Western Blot bands. For qualitative evaluation of the visibility of the test bands (positive versus negative score), RGB pictures of AgPOCT results from randomly chosen replicates were evaluated independently by three individuals in a blinded manner. Furthermore, all test results were scored independently by another person.

### Preparation of test samples from nasopharyngeal swabs

Anonymized, remnant nasopharyngeal swab samples tested positive and negative for SARS-CoV-2 were obtained between May and July 2021 from the Center for Infectious Diseases, Virology, Heidelberg University Hospital, Germany. Samples were stored in VTM. The Ct16, Ct21, and negative test samples were prepared by pooling of 12–15 nasopharyngeal swab samples. Cell debris and other solids were removed by centrifugation at 400*g* for 10 min and subsequent transfer of the supernatant into a new tube. Viral RNA was isolated from pools by manual lysis and automated RNA extraction using the QIAamp Viral RNA Mini kit (Qiagen) on a QIAcube Connect device (Qiagen). Ct values of sample pools were determined by RT-qPCR analysis using the LightMix^Ⓡ^ Modular Sarbecovirus SARS-CoV-2 (TIB Molbiol) with the LightCycler^Ⓡ^ Multiplex RNA Virus Master (Roche) and LightCycler480 II (Roche). Subsequently, pools were supplemented with 2% TritonX-100 and c0mplete Ultra protease inhibitor (Roche) and if needed adjusted with dilution buffer [2 mg/ml BSA, 0.9% NaCl, protease inhibitor]. Ct25 and Ct28 test samples were prepared by dilution of the Ct21 test sample in dilution buffer. Samples were aliquoted (120 µl), immediately frozen on dry ice, and stored at -80 °C. Viral loads of Ct test samples were estimated based on a standard curve determined using the quantitative INSTAND SARS-CoV-2 reference samples 1 and 2 (Ch07469 and CH07470 with defined viral loads of 10,000,000 and 1,000,000 copies/ml; Supplemental Figure S6). For AgPOCT testing, samples were freshly thawed on ice before use. Each time, test samples were validated using a LumiraDx SARS-CoV-2 microfluidics POCT system [6].

### AgPOCTs evaluated in this study

We included a total of 32 AgPOCTs available at local supermarkets, pharmacies, and drugstores as well as on several online trade platforms (Table 1). Specific AgPOCTs will be referred to as the respective manufacturer's name (in italic in Table 1). The inspected AgPOCTs include both, tests for professional in vitro diagnostics use (#1–14) as well as tests temporarily licensed for self-testing in Germany (#15–32) by the Federal Institute for Drugs and Medical Devices [2] (Supplemental Figure S5). The majority of AgPOCTs available were nasal or nasopharyngeal swab tests except BTNX, Ritter, Joinstar, Realy (#11–14) among the tests for professional use and Sanicom, Hygisun, fameditec (#30–32) among the self tests, which are all saliva spit tests, as well as Watmind (#29), which is a saliva swab test.

**Table 1:** AgPOCTs investigated in this study

#	Supplier	AgPOCT name	Specifications	Sample type	Use	Distributor
1	*Abbott* Rapid Diagnostics Jena GmbH	Panbio COVID-19 Ag Rapid test device (nasal)	REF: 41FK11 LOT: 41ADG244A	Nasal swab	pro	Online trade
2	*Healgen* Scientific Limited Liability Company	Coronavirus Ag Rapid Test Cassette (Swab)	REF: GCCOV-502a LOT: 2012650	Nasopharyngeal swab	pro	Online trade
3	*RapiGEN*, INC	Biocredit COVID-19 Ag—one step Rapid Test	REF: G61RHA20 LOT: H073097SD	Nasopharyngeal swab	pro	Online trade
4	Beijing *Beier* Bioengineering Co., Ltd	COVID-19 Antigen Rapid Test Kit	REF: not specified LOT: 20210201	Nasopharyngeal swab	pro	Online trade
5	*möLab* GmbH	mö-screen Testkit Corona Antigen	REF: 0230005B1 LOS: 2104072	Nasal/nasopharyngeal swab	pro	Online trade
6	*Biomerica*, Inc	COVID-19 Antigen Rapid Test	REF: 1509A-25 l LOT: COV6686	Nasopharyngeal swab	pro	Online trade
7	*Joysbio* (Tianjin) Biotechnology Co., Ltd	SARS-CoV-2 Antigen Rapid Test Kit (Colloidal Gold)	REF: G10313 LOT: 2021011607	Nasopharyngeal swab	pro	Online trade
8	*Safecare* Biotech (Hangzhou) Co., Ltd	COVID-19 Antigen Rapid Test Kit (Swab)	REF: COV Ag-6012 LOT: COV21040606	Nasopharyngeal swab	pro	Online trade
9	Hangzhou *Testsea* Biotechnology Co., Ltd	Testsealabs COVID-19 Antigen Test Cassette	REF: 2020013 vB/10 LOT: TL1C05	Nasal swab	pro	Online trade
10	*ACON* Biotech (Hangzhou) Co., Ltd	Flowflex SARS-Cov-2-Antigen-Schnelltest (Selbsttest)	REF: L031-11855 LOT: COV1030052	Nasal swab	pro	Online trade
11	*BTNX* Inc	Rapid Response COVID-19 Antigen Rapid Test Cassette	REF: COV-2C25B LOT: COVG21030089	Saliva (spit)	pro	Online trade
12	Joysbio (Tianjin) Biotechnology Co., Ltd/*Ritter*	Easy Check Spit test SARS-CoV-2 Antigen Rapid Test Kit (Colloidal Gold)	REF: COV-AG-20/G10313 LOT: 20210202	Saliva (spit)	pro	Online trade
13	*Joinstar* Biomedical Technology Co., Ltd	COVID-19 Antigen Rapid Test (Latex)	REF: RPBH12360 LOT: COV2103002L	Saliva/sputum (spit), stool	pro	Online trade
14	Hangzhou *Realy* Tech Co., Ltd	Novel Coronavirus (SARS-CoV-2) Antigen Rapid Test Device (Saliva)	REF: K590516D LOT: 202101022	Saliva (spit)	pro	Online trade
15	*nal von minden* GmbH	NADAL COVID-19 Ag Test	REF: 243103 N-20H LOT: 175363 BfArM GZ: 5640-S-045/21	Nasal swab	lay	Online trade
16	*SD Biosensor*	SARS-CoV-2 Rapid Antigen Test	REF: 9901-NCOV-01G LOT: QCO390092I BfArM GZ: 5640-S-025/21	Nasal swab	lay	Online trade
17	Beijing *Hotgen* Biotech Co., Ltd	Coronavirus (2019-nCoV)-Antigentest	REF: 4260220532859 LOT: W2021032500/602/1500 BfArM GZ: 5640-S-057/21	Nasal swab	lay	Supermarket Pharmacy
18	Guangzhou *Wondfo* Biotech Co., Ltd	2019-nCoV Antigen Test (Lateral Flow Method)	REF: W634P0021 LOT: W634104116 BfArM GZ: 5640-S-179/21	Nasal swab	lay	Supermarket
19	*Teda* Laukoetter Technology GmbH	COVID-19 Antigen Schnelltest (kolloidales Gold) ANBIO Corona Antigen Nasentupfer	REF: A6061214 LOT: 2021046133/461310/036138 BfArM GZ: 5640-S-079/21	Nasal swab	lay	Drug store
20	Beijing *Lepu medical* Technology Co., Ltd	NASOCHECKcomofort SARS-CoV-2 Antigen-Schnelltest	REF: CG2701N LOT: 21CG2720X/18X BfArM GZ: 5640-S-104/21	Nasal swab	lay	Drug store
21	Hangzhou *Clongene* Biotech Co., Ltd	COVID-19 Antigen Rapid Test	REF 6950921302636 LOT: 2021030161 BfArM GZ: 5640-S-168/21	Nasal swab	lay	Online trade
22	Hangzhou *Laihe* Biotech Co., Ltd	LYHER Novel Coronavirus (COVID-19) Antigen Test Kit (Colloidal Gold) NASAL	REF: 303036 LOT: 2103049/47/89-01 BfArM GZ: 5640-S-009/21	Nasal swab	lay	Pharmacy
23	*MP* Biomedicals Germany GmbH	Rapid SARS-Cov-2 Antigen Test Card	REF: 07AG6001BS LOT: 21033003 BrArM GZ: 5640-S-076/21	Nasal swab	lay	Supermarket
24	Xiamen *Boson* Biotech Co., Ltd	Rapid SARS-CoV-2 Antigen Test Card	REF: 1N40C5-4 LOT: 21040609 BfArM GZ: 5640-S-007/21	Nasal swab	lay	Supermarket
25	*NanoRepro* AG	VIROMED for the detection of SARS-Cov-2 from anterior nasal swab	REF: B60500 LOT: 20210401B BrArM GZ: 5640-S-096/21	Nasal swab	lay	Drug store
26	Anhui *Deepblue* Medical Technology Co., Ltd	COVID-19 (SARS-CoV-2) Antigentestkit (kolloidales Gold)	REF: SL030101N-5 LOT: ST210405 BfArM GZ: 5640-S-086/21	Nasal swab	lay	Online trade
27	*OFM* GmbH	Deni COVID-19 Antigen Test—Selbsttest für ZuHause	REF: OFM-LSYBT-NS-1 LOT: P202103003 BfArM GZ: 5640-S-140/21	Nasal swab	lay	Drug store
28	*Medice* Arzneimittel Pütter GmbH & Co. KG	Medicovid-AG SARS-CoV-2 Antigen Selbsttest 5 NASE	REF: 1N40C5-4 LOT: 21041002 BfArM GZ: 5640-S-128/21	Nasal swab	lay	Online trade
29	Shenzhen *Watmind* Medical Co., Ltd	SARS-CoV-2 Antigen Schnelltest zur Eigenanwendung (kolloidales Gold)	REF: LFA0401-5 N LOT: 21040904/21040704 BfArM GZ: 5640–032/21	Saliva (swab)	lay	Supermarket
30	MR *Sanicom* GmbH	COVID-19 Antigen Schnelltest zur Eigenanwendung (Speichel-/Spucktest)	Barcode no: 4260729310002 LOT: CAG2104021G BfArM GZ: 5640-S-147/21	Saliva (spit)	lay	Drug store
31	*Hygisun* Anbio (Xiamen) Biotechnology Co., Ltd	COVID-19 Antigen Schnelltest (kolloidales Gold)	REF: A6061213 LOT: 2021046132/2021036136/2021036137 BfArM GZ: 5640-S-058/21	Saliva (spit)	lay	Drug store
32	*fameditec*	CORA Check-19 Comfort	REF: K590516D/ LOT: 2021022019 BfArM GZ: 5640-S-154/21	Saliva (collected with sponge)	lay	Online trade

For each AgPOCT supplier, name, reference, and LOT number are indicated. If tests obtained a temporary license for self-testing in Germany the corresponding BfArM GZ number is given as well. In addition, sample type and professional (pro) versus layman (lay) use is indicated. In the last column, the type of distributor where AgPOCTs were purchased is noted

For Lepu medical (#20 in Table 1), the AgPOCT with the poorest results in our study, we purchased different versions and additional batches for in-depth characterization (Table 2).

**Table 2:** Lepu medical AgPOCT products investigated in this study

Product name	Packaging size (use)	Specifications	Production and use-by date
SARS-CoV-2 Antigen Rapid Test Kit (Colloidal Gold Immunochromatography) CE	Pack of 25 tests/(pro)	Barcode: 6921807601020 LOT: 21CG2713X	Production date: 06/03/2021 Use-by date: 06/03/2022
		Barcode: same as above LOT: 21CG2715X	Production date: 03/11/2021 Use-by date: 03/11/2022
NASOCHECKcomfort SARS-CoV-2 Antigen Schnelltest Immunchromatographischer Test (kolloidales Gold). Schnell & angenehm! Einfache Anwendung im vorderen Nasenbereich	Pack of 25 testes (lay)	REF: CG2725(N) Barcode: 4260716970042 LOT: 21CG2722X BfArM GZ: 5640-S-104/21	Production date: 31/03/202 Use-by date: 31/03/2022
NASOCHECKcomfort SARS-CoV-2 Antigen-Schnelltest. Zur Eigenanwendung. Schnell & angenehm! Einfache Anwendung im vorderen Nasenbereich	Single pack (narrow) (lay)	REF: CG2701N Barcode: 4260716970059 LOT: 21CG2727X BfArM GZ: 5640-S-104/21	Production date: 11/04/2021 Use-by date: 11/04/2022
NASOCHECKcomfort SARS-CoV-2 Antigen-Schnelltest. Zur Eigenanwendung. Schnell & angenehm! Einfache Anwendung im vorderen Nasenbereich	Single pack (thick) (lay)	REF: CG2701N Barcode: 4260716970059 LOT: 21CG2720X BfArM GZ: 5640-S-104/21	Production date: 26/03/2021 Use-by date: 26/03/2022
		REF, barcode, BfArM GZ: same as above LOT: 21CG2724X	Production date: 04/04/2021 Use-by date: 04/04/2022

AgPOCT products made by Beijing Lepu Medical Technology Co., Ltd. are referred to as Lepu medical AgPOCTs. Lepu medical AgPOCTs were purchased on different online trade platforms. For each Lepu medical AgPOCT, product name, packaging size, and intended use (professional (pro) versus layman (lay) use), reference/barcode number, LOT number as well as BfArM GZ number if applicable are indicated. Production and use-by date are noted in the last column

## Results

### Generation of test samples for standardized AgPOCT evaluation

In the present study, we sought to establish a standardized procedure to rapidly assess the sensitivities of a large number of SARS-COV-2 AgPOCTs. To this end, we generated a collection of test samples from pooled nasopharyngeal swabs from SARS-CoV-2 positive-tested and negative-tested individuals. Ct values of the SARS-CoV-2 positive pools were determined by RT-qPCR and test samples were prepared accordingly. The test sample collection comprised four SARS-CoV-2 positive pools with defined viral loads (Ct16, Ct21, Ct25, and Ct28) and one SARS-CoV-2 negative pool. The positive pools covered high (Ct values < Ct24) and moderate (Ct values between Ct24 and Ct30) loads of SARS-CoV-2 genetic material. Per test sample, > 200 aliquots with 120 µł sample volume each were prepared, allowing a quick and standardized evaluation of the analytical sensitivities of a large number of different AgPOCTs.

We estimated that our test sample collection covers a range from 9.8 × 10^8^ (Ct16) to 1.8 × 10^5^ (Ct28) SARS-CoV-2 genome copies per ml (Supplemental Figure S6). We qualitatively validated the test sample collection using a LumiraDx Covid-19 antigen test device. This microfluidic POCT system was manufactured in Europe and able to detect the viral antigens in the most diluted sample. This is consistent with previous reports on the high sensitivity of the LumiraDx device [6]. We used 50 µl of a test sample, each for the LumiraDx analysis and for all AgPOCTs evaluated in this study, as described before [7, 8]. All four SARS-CoV-2 positive test samples tested positive for SARS-CoV-2, while the negative test sample was recognized as negative in the LumiraDx analysis.

### Quantitative and qualitative assessment of AgPOCT analytical sensitivity

**Fig. 1:**
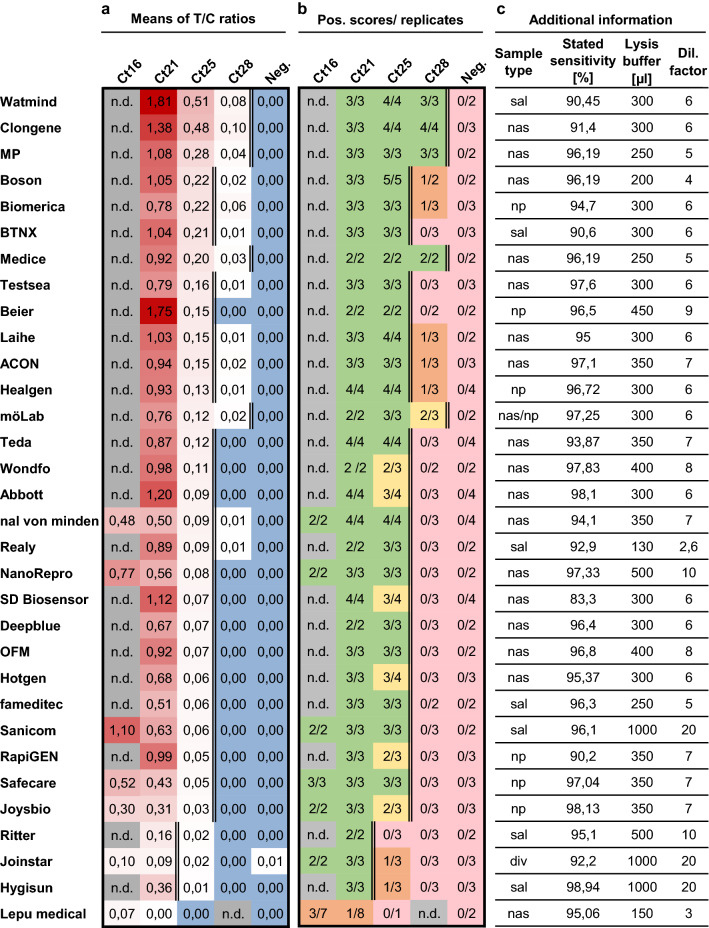
Quantitative and qualitative evaluation of 32 SARS-CoV-2 AgPOCTs in a rapid sensitivity assessment approach. **a** Investigated AgPOCT are listed with the means of *T*/*C* ratios (test band (*T*) intensity to control (*C*) band intensity) for each Ct test sample. *T*/*C* ratios are color-coded in shades of red (highest values with most intense color). Blue color highlights zeros indicating the absence of a measurable signal at the test band position. Ct16 test sample was only used on AgPOCTs with exceptionally low performance in detection of the Ct21 sample. AgPOCTs are ranked according to their *T*/*C*_Ct25_ ratio. **b** Scoring results of visual inspection for all replicates. Full reproducibility of positive scores in all replicates is highlighted in green, positive scores in the majority of replicates in yellow, positive scores in the minority of replicates in orange, and no positive scores in any replicate in light red. n.d. = not determined (grey). A double line indicates the limit of reliable detection of SARS-CoV-2 positive samples (reliability defined by the reproducibility of positive scores in all (green) or most (yellow) replicates of a given Ct test sample). **c** Additional information on investigated AgPOCTs: Sample type (nasal (nas)/nasopharyngeal (np) swab, saliva (sal) or diverse (div)), sensitivities of AgPOCTs according to the corresponding manufacturer’s package insert, volumes of provided lysis buffers, and the resulting dilution factor for the Ct test samples (*V* = 50 µl) are given

We tested a total of 32 AgPOCTs (Table 1). We purchased 12 AgPOCTs from local resellers (pharmacies, drugstores, supermarkets) and 20 products online. We performed the tests over 10 days, with the help of four students, during the course of four weeks. For each product, we used freshly thawed aliquots of the Ct21, Ct25, and Ct28 test samples as well as the negative sample. We conducted two to four replicates per product and acquired images of each of the tests at the time points specified by the manufacturers. The Ct16 test sample was only used for AgPOCTs that had a low performance with the Ct21 test sample. For quantitative evaluation, signal intensities of the test (*T*) and the control (*C*) bands were measured and the ratio of these values (*T*/*C* ratio) was determined (Fig. 1a). In addition, we scored a binary (positive or negative) test result using visual inspection of the images by four different persons (Fig. 1b).

**Fig. 2:**
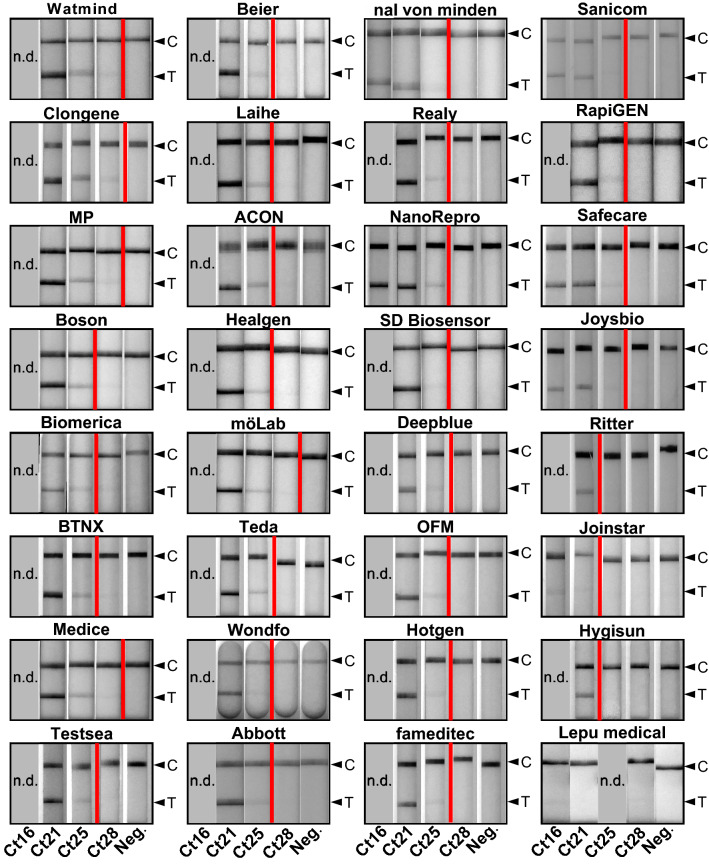
Representative images of SARS-CoV-2 AgPOCTs lateral flow test stripes treated with corresponding Ct test samples. Contrast settings were optimized for each AgPOCT example image set to ensure the best visibility of the test bands. AgPOCT example images are arranged (from top left to bottom right) according to the ranking presented in Fig. 1. The red line indicates the limit of reliable detection (see Fig. 1a, b). Arrowheads highlight positions of control (*C*) and test (*T*) bands

For 31 of 32 investigated AgPOCTs, an average *T*/*C*_Ct25_ > 0 was determined for all positive test samples and not for the negative control sample (Fig. 1a). This indicates that the digital quantification detects test band signals for 31 AgPOCTs using the Ct25 test sample, albeit some test bands had extremely weak signal intensities (Fig. 2). For almost all AgPOCTs, no signal could be measured when the SARS-CoV-2 negative test sample was applied. Only for Jointstar, one replicate of the negative test sample resulted in a false positive test band indicated by a *T*/*C*_Neg._ > 0. In contrast to the more sensitive digital quantification, the visual inspection did only score a positive result for 28 of 31 AgPOCTs with a *T*/*C*_Ct25_ > 0 (Fig. 1b, Fig. 2). This is also true for the visual assessment of the results of technical replicates. For example, for Jointstar, the negative sample with a *T*/*C*_Neg._ > 0 scored negative in the visual inspection. We could not establish a specific *T*/*C* value threshold to explain the results of the visual assessment, indicating that these ratios are product-specific. This can be explained by different dyes, and by the fact that the visual assessment was conducted using color vision, while for the *T*/*C* quantification grayscale images were used. Furthermore, we observed large coefficients of variation (CV) for some of the tests, in particular for samples with very small *T*/*C* ratios, emphasizing weak signals close to the detection limit of the digital quantification (Supplemental Figure S1c).

We grouped the tested AgPOCTs into categories with low (Group III), medium (Group II), and high (Group I) sensitivity based on the reliability to detect a given SARS-CoV-2 positive sample. A sample was considered reliably detected by a given AgPOCT when all or the majority of replicates (at least two out of three or three out of four replicates) of a given sample were scored positive. If none or the minority of replicates of a given sample was detected by the corresponding AgPOCT, reliability requirements were not met.

One exception was Lepu medical (Table 1, AgPOCT #20; Table 2, last row), which did not fulfill the requirements for any of these groups. For Group III AgPOCTs with the lowest sensitivity, the minimum criterion was the reliable detection of the Ct21 sample. Lepu medical tests failed this, as these did not even reliably score a positive result with the Ct16 sample (9.8 × 10^8^ copies/ml; Fig. 1b, Fig. 2). To investigate this product further, we used individual unprocessed nasal/nasopharyngeal swab samples with low Ct values (Ct13.3 to Ct18.4) on this and another poor performing product. Comparison of these results to the Ct16 test sample confirmed the low sensitivity of the Lepu medical test (Supplemental Figure S2). Only for samples with very low Ct values (Ct∼13), *T*/*C* ratios were obtained that can be detected easily visually (Supplemental Figure S2a). This suggests that this product is not completely non-functional, but largely insensitive. Since Lepu medical AgPOCTs have been widely used in Germany and other European countries we retrieved several Lepu medical products available on different online trade platforms (Table 2). These included two different batches of a Lepu medical product intended for professional use (Table 2, first row, no BfArM GZ number, CE mark). Additionally, we included different batches and deviations of the Lepu medical AgPOCT described before (Table 1, AgPOCT #20; Table 2, last three rows). These products were provided with the same BfArM GZ number (5640-S-104/21), which cannot be found on the BfArM list anymore (Supplemental Figure S5). We investigated their performance in direct comparison in multiple replicates using Ct16, Ct21, and Ct25 test samples (Supplemental Figure S3). This revealed high variation of the determined T/C ratios, with coefficients of variation (CV) ranging from 0.26 to 1.54 and a median CV of 0.50 (Supplemental Figure S4c). In contrast, the median CV of the other 31 investigated AgPOCTs with Ct16-25 test samples was 0.11 (Supplemental Figure S1c). This indicates a larger fluctuation of the test results not only for different implementations of the Lepu medical AgPOCTs but also for different batches of the same Lepu medical product, compared to all other AgPOCTs investigated in this study.

AgPOCTs in Group III only reliably detected the Ct21 sample (2.7 × 10^7^ copies/ml) and included Hygisun, Joinstar, and Ritter. Of note is that all of them are saliva-based spit tests (Table 1), which are provided with a considerably larger amount of lysis buffer (500–1000 µl lysis buffer; Fig. 1c) than most other AgPOCTs. This results in an increased dilution of the test sample compared to AgPOCTs for nasal samples, which are provided on average with 320 µl lysis buffer (Fig. 1c). The higher dilution of the sample together with the possibility of lower virus concentration in saliva versus nasal or nasopharyngeal swabs may further influence the analytical sensitivity of these AgPOCTs.

The large majority of the investigated AgPOCTs (23 out of 32) delivered visible positive results with the Ct25 sample (1.6 × 10^6^ copies/ml, Group II). Among these 23 AgPOCTs, positive scoring was fully reproducible in all replicates for 17 AgPOCTs. AgPOCTs intended for professional use (sorted ascending according to T/C_Ct25_: Safecare, Realy, Healgen, ACON, Beier, Testsea, BTNX, and Biomerica) largely cluster in the upper half of the T/C_Ct25_ ranking, while tests licensed for self-testing largely cluster in the lower half (sorted ascending according to T/C_Ct25_: Sanicom, fameditec, OFM, Deepblue, NanoRepro, nal von minden, Teda, Laihe and Boson). Interestingly, among both tests for professional and for layman use, saliva spit tests (Realy, Sanicom, fameditec) appear largely inferior compared to nasal swab tests in this setting except for of BTNX, which is the sixth-highest ranked AgPOCT among all investigated tests. Six AgPOCTs in Group II (sorted ascending according to *T*/*C*_Ct25_: Joysbio, RapiGEN, Hotgen, SD Biosensor, Abbott, and Wondfo) failed in one out of three to four replicates to detect the Ct25 sample, which is represented by larger CV values ranging from 0.26 to 0.87 (Supplemental Figure S1c).

Using the Ct28 test sample (1.8 × 10^5^ copies/ml), 14 out of 32 AgPOCTs yielded a *T*/*C*_Ct28_ > 0; however, only five reliably scored a positive result in the visual investigation (Group I). These include (sorted in ascending order according to T/C_Ct25_) möLab, Medice, MP, Clongene, and Watmind, three of which are temporarily licensed for self-testing (Table 1, Supplemental Figure S5). All, except möLab, delivered a positive visual result in all three replicates.

Taken together, the data presented here demonstrate that the different SARS-CoV-2 AgPOCTs available deviate largely in the analytical sensitivity of the lateral flow test stripes and provided buffer systems, corresponding more than two orders of magnitude of viral genome copies per ml (9.8 × 10^8^ to 1.8 × 10^5^). Additionally, we revealed great variation in results delivered with different Lepu medical AgPOCT versions and batches emphasizing the need for regular quality monitoring.

## Discussion

Currently, there are more than 500 different products available for SARS-CoV-2 diagnostics, many of which lack an independent assessment of their performance. In most cases, the clinical sensitivity values provided by the manufacturer (examples in Fig. 1c) are far > 90%. However, detailed information on specimen collection and viral loads are usually not provided rendering these values largely inconclusive and misleading for laymen. Considering that individual products use different antibodies in varying amounts with different specificities and affinities sometimes recognizing different proteins in the viral particle with unequal abundances, and diverse staining methods, these conspicuously similar values for clinical sensitivity given by the manufacturers are also unlikely. Therefore, an independent, rapid and critical evaluation of AgPOCTs available is required to determine the realistic performance of AgPOCT relevant to the daily user and especially to identify poor performing products. Given the huge number of products available for rapid SARS-CoV-2 diagnostics, in-depth studies evaluating the quality of AgPOCTs in a time-intensive procedure will not be available any time soon for all products available.

We developed a straightforward strategy to quickly evaluate the technical sensitivity of AgPOCTs for SARS-CoV-2. We generated four SARS-CoV-2 positive reference samples by pooling several individual samples with high viral loads followed by RT-qPCR analysis and subsequent adjustment of the pools to the desired Ct values with buffer. Reference samples with higher Ct values were generated by further dilution of the above-mentioned pools. This approach results in mixed samples and the impact of contaminations in individual samples needs to be considered: potentially, inhibitory contaminations or contaminations that are causing false-positive results may be present in one or the other sample used for the pools. However, such samples are usually an exception. Furthermore, the pooling and Ct adjustment procedure results in a 15- to 20-fold dilution of such constituents so that their impact is reduced if not completely marginalized. Nevertheless, one needs to consider that individual AGPOCTs may be influence by such contaminations. The reference samples were adjusted to span the relevant dynamic range of the typical sensitivity of AgPOCTs ranging from Ct28 to Ct16 (corresponding to approximately 1.8 × 10^5^ to 9.8 × 10^8^ SARS-CoV-2 genome copies per ml). Lower Ct values correspond to higher viral loads. The Ct value alone cannot inform about the fraction of viable virus; however, multiple studies showed that high viral loads (Ct values < Ct24) upon detection of a SARS-CoV-2 infection relate to higher morbidity and infectivity potential (summarized in [9]). Therefore, AgPOCTs are, despite their inferior sensitivity compared to laborious RT-qPCR-based SARS-CoV-2 diagnostics, a critical tool in aiding disease prognosis and viral containment by detecting these high to moderate viral loads.

Using these reference samples, we were able to group 32 commercially available products into AgPOCT groups with high, average and low sensitivity (Group I-III). Most importantly, we identified one product that did not detect any of the test samples and, therefore, is considered not suitable for SARS-CoV-2 diagnostics.

The majority of tests investigated in this study reliably detected the Ct25 test sample as SARS-CoV-2 positive (Group II). Some of these AgPOCTs have been thoroughly characterized, including Abbott, RapiGEN, Healgen, nal von minden, and SD Biosensor by Corman et al. [7] and others [4, 8–23]. Corman et al. determined 95% limits of detection for each AgPOCT using 138 SARS-CoV-2 positive clinical samples with viral loads ranging from 1.9 × 10^4^ to 2.8 × 10^9^ genome copies per ml. Among the AgPOCTs also tested in this study, Healgen was most sensitive, followed by Abbott, SD Biosensor, and nal von minden—all with a 95% limit of detection between 2.3 and 9.3 × 10^6^ SARS-COV-2 genomes per swab. In contrast, for RapiGEN, a 95% limit of detection more than three orders of magnitudes lower was found. This discrepancy in performance between RapiGEN and the above-mentioned products is supported by other studies [24]. In our analysis, this trend is reflected as well even though we cannot resolve the limits of detection in such great detail: For Healgen and nal von minden, detection of the Ct25 test sample (1.6 × 10^6^ copies/ml) was robust with all replicates being positively scored. For RapiGEN, Ct25 test sample detection was less reliable, and based on the *T*/*C*_Ct25_ this product is ranked in the lowest quarter among all AgPOCTs investigated.

Among the 32 investigated AgPOCTs, we identified four reliably well-performing AgPOCTs, which detected the Ct28 test sample (1.8 × 10^5^ copies/ml) as SARS-CoV-2 positive in all replicates (Group I). Amongst these, Watmind was ranked highest in our evaluation. This AgPOCT is also among the best-performing three AgPOCTs out of 122 tested products with a sensitivity of 82% in samples with Ct values ranging from Ct17 to Ct35 corresponding to viral loads of > 10^8^ to 10^3^ SARS-CoV-2 genome copies per ml [4].

Group III includes AgPOCTs with lower analytical sensitivity as these only detected the Ct21 test sample as SARS-CoV-2 positive. For Joinstar, using Latex beads for visualization, evidence provided by Scheiblauer and colleagues [4] suggests that this test is non-functional with 0% sensitivity for all sample panels supporting the low ranking of Joinstar in this study. In our analysis, we detected weak bands for the Ct21 and Ct16 test samples; however, these were considerably weaker than for all other tests suggesting the possibility that latex beads used for visualization do fail to produce a strong signal. Besides Joinstar, Ritter andHygisun, both saliva spit tests as well, showed low sensitivities in our studies. While we could not find independent evaluation studies for these products, both can be found on the BfArM list (as of July 23, 2021; Supplemental Figure S5).

Among the low ranked AgPOCTs, the sensitivity of the Lepu medical AgPOCT was exceptionally low as this test failed to deliver a visible positive test result in most replicates, even for the Ct16 sample. In addition to its poor performance in SARS-CoV-2 diagnostics, out of 20 performed Lepu medical tests, three tests technically failed, indicated by the absence of the control band. Importantly, this AgPOCT is a popular product in the area where this study was conducted and is still available at many drugstores and supermarket chains. Of note is that Lepu medical differs from other AgPOCTs in its design and sample application. Technical failure did not occur in any of the other AgPOCTs, in which the immunochromatography paper is embedded in the common plastic cassette. In other studies, Lepu medical AgPOCT products performed better [4, 25], e.g. in the setting of Scheiblauer et al*.* [4], a sensitivity of 100% was found for a Lepu medical AgPOCT and test panel members with Ct values ranging from Ct17 to Ct25. As the AgPOCTs used in these studies are not specified with reference/product and LOT number, it is possible that a different Lepu medical product or batch was used. We purchased different Lepu medical AgPOCT products available online and compared performances on the pooled test samples and raw, unprocessed swab samples (Supplemental Figure 3). We tested two batches of a CE-marked product and four Lepu medical with the same BfArM GZ number, but different packaging versions (Table 2). Indeed, we identified two Lepu medical products performing better than shown in Fig. 1; however, these performances were not reproducible with other batches of the same product (Supplemental Figure 3), indicating batch-specific variation of the quality. Issues regarding the quality of rapid AgPOCTs are reported not only for SARS-CoV-2 [1], but also other infectious diseases such as malaria [26]. This, again, emphasizes the importance of a simple method to assay the performance of an AgPOCT product and corresponding batches. In summary, a comparison with published data for some of the investigated products confirmed our results. Therefore, we provide evidence that our chosen strategy constitutes a viable solution to rapidly assess the sensitivity of SARS-CoV-2 AgPOCTs.

It is important to mention that the sensitivity of an AgPOCT is the product of multiple factors; the sensitivity of the test stripe and buffer system used are important contributing factors, but not the only ones. Another is the volume of the lysis buffer provided with each AgPOCT. The volume varies between tests of different manufacturers resulting in a 2.6- to 20-fold dilution of the samples (Fig. 1c). In this study, we did not correct the test results for these different dilution factors, because the sample dilution is an internal property of each AgPOCT. By using the same sample volume for each AgPOCT we also neglect potential differences in swab properties, such as absorption volume or sample specimen (saliva, nasal or nasopharyngeal samples), which affect the diagnostic sensitivity of AgPOCTs. However, we note that for tests based on nasal swabs the used volume of 50 μl approximates the quantity absorbed by these swabs [7]. Furthermore, the AgPOCT-specific instructions for self-sampling, which will influence how carefully a sample is collected, can also influence the diagnostic sensitivity of a test. In light of these considerations, we want to emphasize that this evaluation method only and exclusively focuses on comparing the technical sensitivity of the lateral flow test strips from different test manufacturers, in combination with the provided lysis buffers.

The procedure presented here involving a reduced test sample collection and minimal labor represents a feasible strategy for prompt evaluation of available AgPOCTs for their usability in SARS-CoV-2 diagnostics. We provide a useful estimation of the limits of detection for the investigated AgPOCTs as the dimensions and trends are comparable to results from much more laborious in-depth studies. Importantly, using this approach, we revealed very heterogeneous results for the Lepu medical AgPOCT, which precludes in our opinion the use of this product (or product family) for SARS-CoV-2 diagnostics. In conclusion, we suggest this procedure as a rapid alternative to investigate COVID-19 AgPOCTs in the absence of reliable data that validate the performance of a specific product and related batches.

## Supplementary Information

Below is the link to the electronic supplementary material.

Supplementary file1 (DOCX 1944 KB)
