# Supplementary material for: Rapid comparative evaluation of SARS-CoV-2 rapid point-of-care antigen tests
Source: Infection. 2022 Apr 9;50(5):1281–93. doi: 10.1007/s15010-022-01810-1 (PMC8994089; doi:10.1007/s15010-022-01810-1)
Supplement: Supplementary file 1 — Supplementary file1 (DOCX 1944 KB) [file 15010_2022_1810_MOESM1_ESM.docx]

**Supplemental material**

**
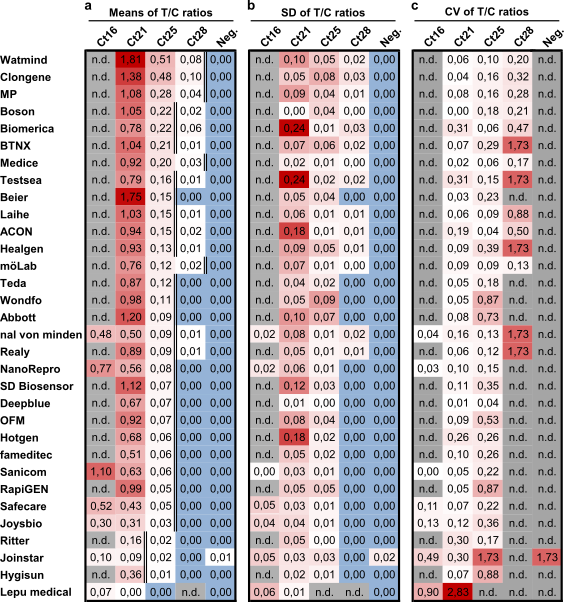
**

**Supplemental Figure S1: Color-coded representation of the standard deviation (SD) and coefficient of variation (CV) calculated for the T/C ratios of different AgPOCTs treated with different Ct test samples.** (a) Means of T/C ratios (test band (T) intensity to control (C) band intensity) for each AgPOCT and Ct test sample (reproduced from Figure 1). The double line indicates the limit of reliable detection of SARS-CoV-2 positive samples (see Figure 1a, b). (b) Standard deviations (SD) of T/C ratios for each AgPOCT and Ct test sample. (c) Coefficients of variation (CV) of T/C ratios for each AgPOCT and Ct test sample. Means, SD, and CV are color-coded in shades of red (highest values with most intense color). Blue color highlights zeros. n.d. (grey) = not determined.

**
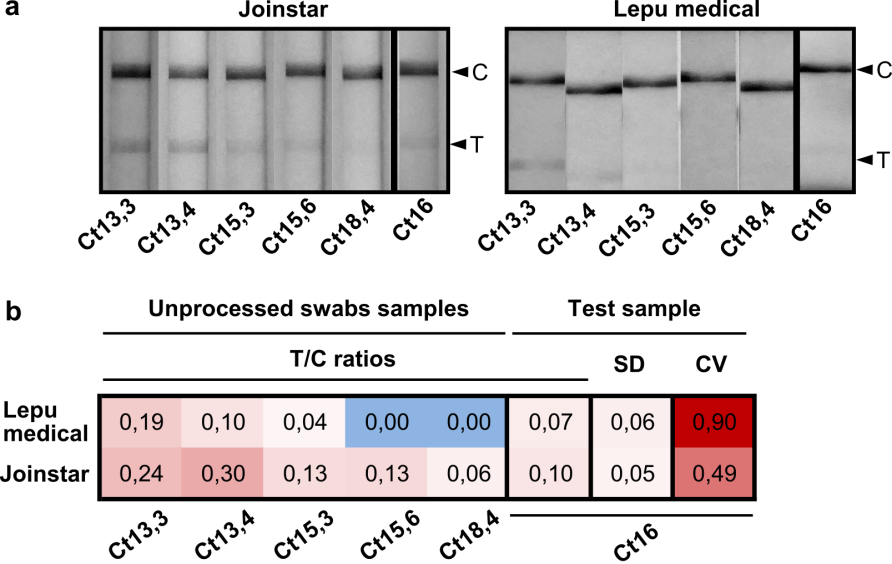
**

**Supplemental Figure S2: Testing of individual, unprocessed nasopharyngeal swab samples on low-performing AgPOCTs.** (a) Representative images of lateral flow test stripes of Lepu medical and Joinstar with unprocessed and Ct16 test samples. Arrowheads highlight positions of control (C) and test (T) bands. The thick black line separates unprocessed samples from the Ct16 test sample. (b) Means of T/C ratios (test band intensity to control band intensity) for Lepu medical and Joinstar with unprocessed swab samples and Ct16 test sample (data for Ct16 test sample reproduced from Figure 1A). Standard deviations (SD) and coefficients of variation (CV) of T/C ratios are given for the Ct16 test sample. Means, SD, and CV are color-coded in shades of red (highest values with most intense color). Blue color highlights zeros. n.d. = not determined (grey).


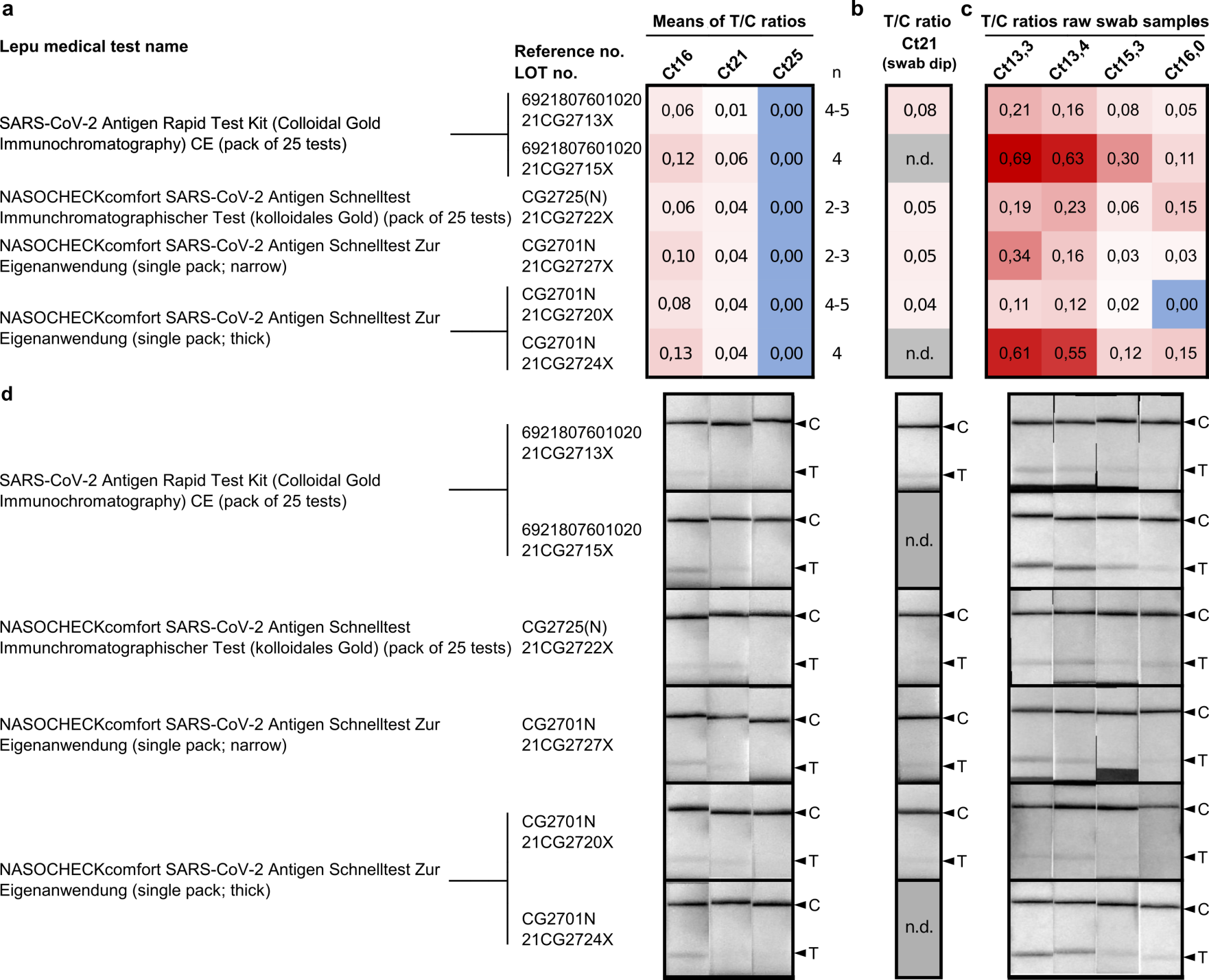


**Supplemental Figure S3: Comparison of different Lepu medical AgPOCT products.** *(continued on next page)*

**Supplemental Figure S3: Comparison of different Lepu medical AgPOCT products.** *(continued)* Different Lepu medical AgPOCT products are listed with reference/ barcode number and LOT number in the first column. The first two products did not have a BfArM GZ number and were CE-marked. The remaining products were provided with the BfArM GZ 5640-S-104/21. (a) Means of T/C ratios (test band (T) intensity to control (C) band intensity) for each Lepu medical AgPOCT product and Ct test sample (Ct16, Ct21, Ct25). Test samples were applied on swabs by pipetting. Numbers of replicates (n) are provided. (b) T/C ratios obtained for each Lepu medical AgPOCT and Ct21 test sample when swabs were dipped into the sample (n=1). (c) T/C ratios for each Lepu medical AgPOCT and unprocessed, raw swab samples in VTM with Ct values ranging from Ct13,3 to Ct16,0 (n=1). Values are color-coded in shades of red (highest values with most intense color). Blue color highlights zeros. n.d. (grey) = not determined. (d) Representative images of lateral flow test stripes of different Lepu medical products and corresponding samples (a-c). Arrowheads highlight positions of control (C) and test (T) bands.

**
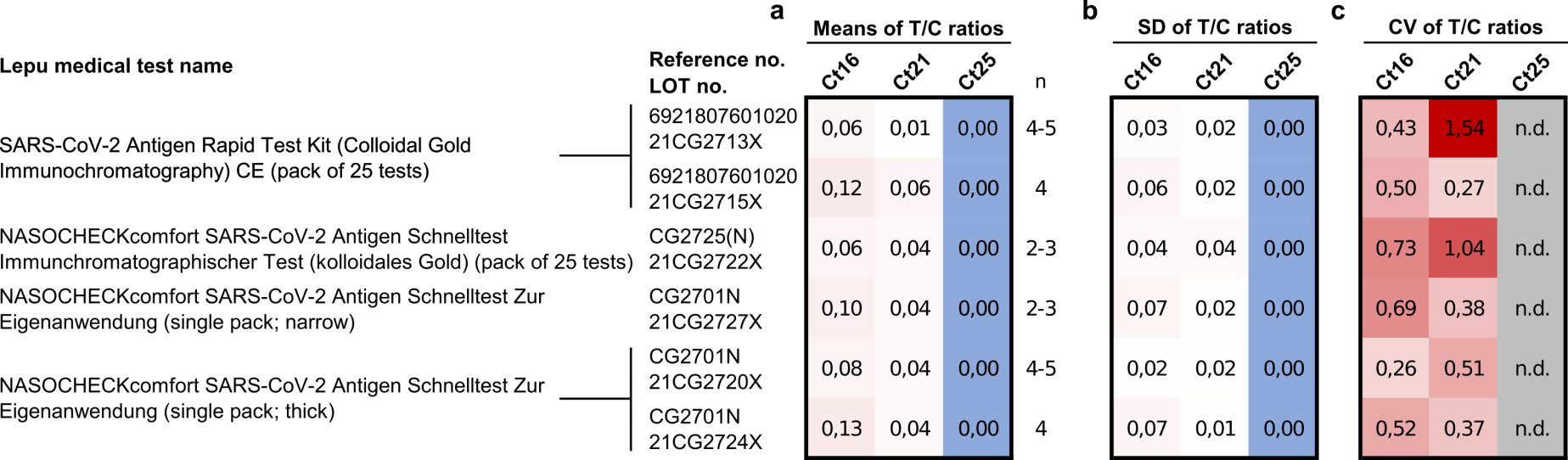
**

**Supplemental Figure S4: Variation in T/C ratios determined for different Lepu medical AgPOCT products.** (a) Means of T/C ratios (test band (T) intensity to control (C) band intensity) for each AgPOCT and Ct test sample (reproduced from Supplemental Figure S3). Numbers of replicates (n) are provided. (b) Standard deviations (SD) of T/C ratios for each Lepu medical AgPOCT product and Ct test sample. (c) Coefficients of variation (CV) of T/C ratios for each Lepu medical AgPOCT product and Ct test sample. Means, SD, and CV are color-coded in shades of red (highest values with most intense color). Blue color highlights zeros. n.d. (grey) = not determined.

**Supplemental Figure S5: Information on BfArM listing and evaluation of investigated AgPOCTs by the Paul Ehrlich Institute** (as of July 27, 2021)**.** AgPOCTs are listed according to the ranking presented in Figure 1. (Middle column) AgPOCTs temporarily licensed for self-testing are provided with the respective BfArM GZ numbers (5640-S-XXX/20 or 21). AgPOCTs for professional use are listed with the corresponding BfArM test ID (ATXXX/ 20 or 21). (Left column) Presence of specified products (according to BfArM number given) on BfArM lists for rapid antigen tests for self-testing or professional use (*) is indicated in light green. Note that if AgPOCTs for professional use are not BfArM-listed (light red; ? in middle and right column), they either failed PEI evaluation or manufacturers did not apply for BfArM listing. If AgPOCTs for self-testing are not BfArM-listed (light red), special permits might have expired or conformity assessments were completed. (Right column) Evaluation of AgPOCTs by PEI according to BfArM lists is indicated in light green. Note that AgPOCTs for self-testing with temporary special permits need a positive evaluation of the product for professional use. Light red indicates missing PEI evaluation.
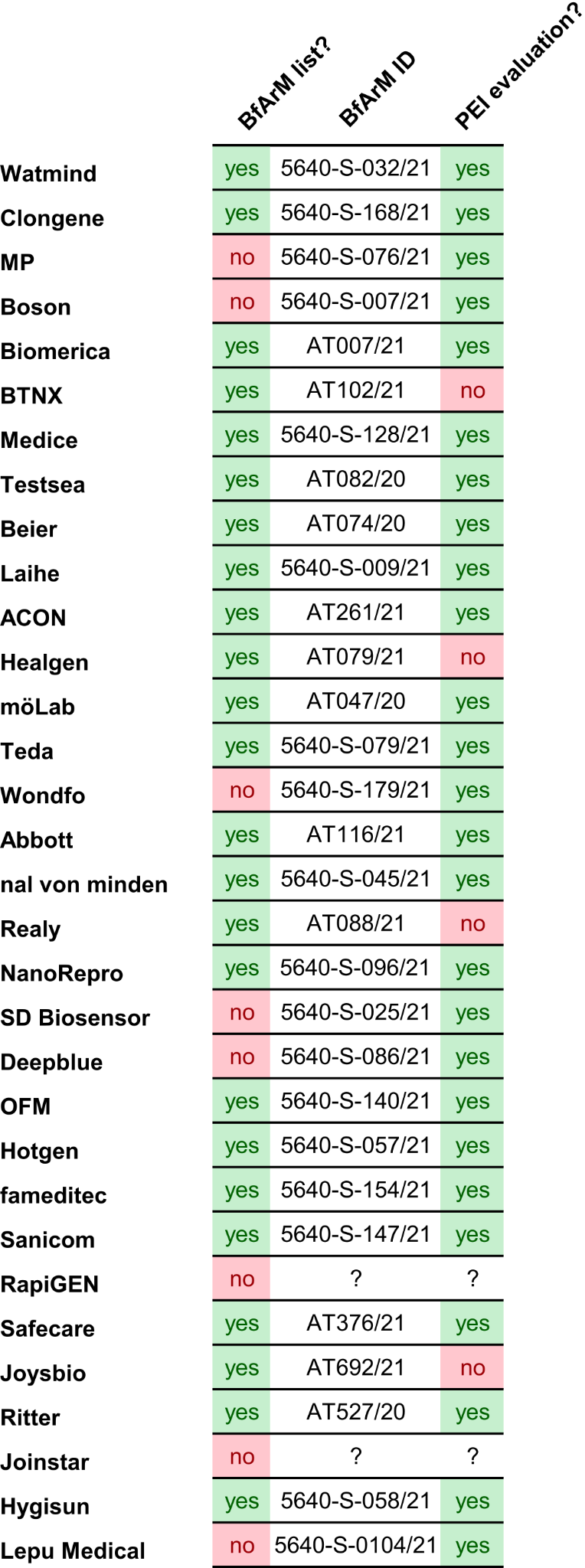


*https://www.bfarm.de/DE/Medizinprodukte/Aufgaben/Spezialthemen/Antigentests/_node.html

**
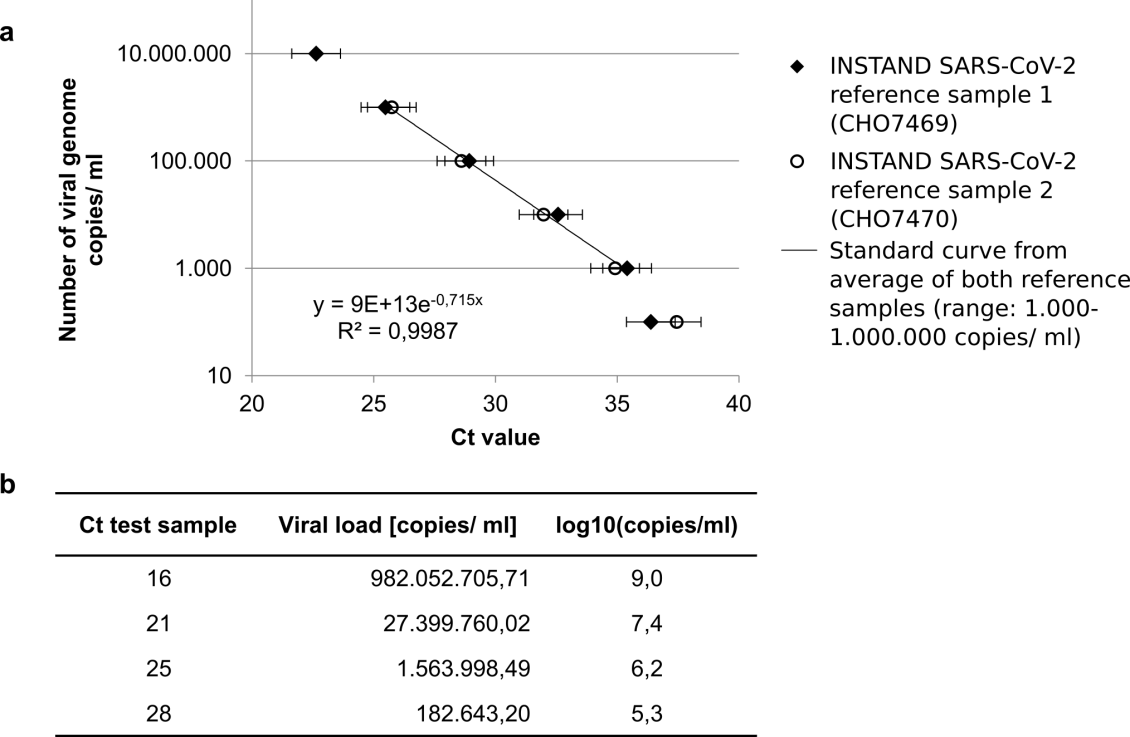
**

**Supplemental Figure S6: Standard curve for estimation of SARS-CoV-2 viral loads in test samples.** (a) Ct values (mean from triplicates ± standard deviation) determined by RT-qPCR for the INSTAND quantitative reference samples 1 (CHO7469; 10.000.000 viral genome copies/ ml) and 2 (CHO7470; 1.000.000 viral genome copies/ ml) and tenfold dilutions thereof. The standard curve was determined based on the average of both reference samples in the range of 1.000-1.000.000 viral genome copies/ ml. The formula as well as the coefficient of determination R² are given. (b) Viral loads calculated for the different test samples based on the standard curve presented in (a).
